# Family members’ experience with in-hospital health care after severe traumatic brain injury: a national multicentre study

**DOI:** 10.1186/s12913-018-3773-7

**Published:** 2018-12-07

**Authors:** Unn Sollid Manskow, Cathrine Arntzen, Elin Damsgård, Mary Braine, Solrun Sigurdardottir, Nada Andelic, Cecilie Røe, Audny Anke

**Affiliations:** 1 Department of Rehabilitation, University Hospital of North Norway, Tromsø, Norway; 2 Faculty of Health Sciences, Department of Health and Care Sciences, UiT, The Arctic University of Norway, Tromsø, Norway; 3 Faculty of Health Sciences, Department of Clinical Medicine, The Arctic University of Norway, Tromsø, Norway; 4 School of Health and Society, University of Salford, Salford, UK; 5 Department of Research, Sunnaas Rehabilitation Hospital, Nesoddtangen, Norway; 6 Department of Physical Medicine and Rehabilitation, Oslo University Hospital, Oslo, Norway; 7 Institute of Health and Society, Research Centre for Habilitation and Rehabilitation Models and Services (CHARM), Faculty of Medicine, University of Oslo, Oslo, Norway

**Keywords:** Experience of care, Quality of care, Traumatic brain injury, Rehabilitation, Family

## Abstract

**Background:**

Family member’s experience and satisfaction of health care in the acute care and in-patient rehabilitation are important indicators of the quality of health care services provided to patients with severe traumatic brain injury (TBI). The objective was to assess family members’ experience of the health care provided in-hospital to patients with severe TBI, to relate experiences to family member and patient demographics, patients’ function and rehabilitation pathways.

**Methods:**

Prospective national multicentre study of 122 family members of patients with severe TBI. The family experience of care questionnaire in severe traumatic brain injury (FECQ-TBI) was applied. Independent sample t-tests or analysis of variance (ANOVA) were used to compare the means between 2 or more groups. Paired samples t-tests were used to investigate differences between experience in the acute and rehabilitation phases.

**Results:**

Best family members` experience were found regarding information during the acute phase, poorest scores were related to discharge. A significantly better care experience was reported in the acute phase compared with the rehabilitation phase (*p* < 0.05). Worst family members` experience was related to information about consequences of the injury. Patient’s dependency level (*p* < 0.05) and transferral to non-specialized rehabilitation were related to a worse family members` experience (*p* < 0.01).

**Conclusions:**

This study underscores the need of better information to family members of patients with severe TBI in the rehabilitation as well as the discharge phase. The results may be important to improve the services provided to family members and individuals with severe TBI.

## Background

Severe traumatic brain injury (TBI) is internationally recognized as a major public health problem causing death and disability [1–3]. For survivors of severe TBI, the most common disabilities are associated with cognitive and behavioural deficits, which can impact the injured, caregivers and other family members for a long period of time [4, 5]. Individuals with severe TBI may face a long in-hospital stay during both the acute and rehabilitation phases. To achieve optimal outcomes, complex medical, nursing and rehabilitative care is required [6].

Family members’ experience and satisfaction with the health care services is an important indicator of the quality of care provided [7–9]. In fact, experiences of family members are key dimensions of health care quality, as they often act as the patient’s representative and play a key role in care and support [10]. The two concepts of experience and satisfaction are used interchangeably in the literature. However, experience relates more specifically to detailed experiences with the health care services, whereas satisfaction is often assessed as a global measure, sometimes via only one item. Exploring actual experiences with health care generally results in less positive findings [11].

There is a lack of longitudinal studies investigating family members’ experiences during the different phases of in-hospital TBI treatment from the acute care, through rehabilitation and until discharge home. A qualitative study found that lack of information and education before discharge and a lack of preparation for the future were prominent themes [12]. In addition, these researchers reported that this generated a considerable uncertainty regarding the patient’s transition between the in-hospital acute and rehabilitation phases [12]. Two studies have revealed that family members need more information about the nature and consequences of TBI in both the acute and rehabilitation phase to be able to understand the consequences of the injury as well as be prepared for the future [13, 14].

Only two TBI studies have explored family members’ experiences in the phase from in-patient rehabilitation until discharge to home [15, 16]. The study of Nalder and colleagues (2012) investigated factors associated with perceived success in the transition from hospital to home. They found that lower ratings of transition success were associated with greater stress among family members [16]. The other study by O’Callaghan and colleagues (2011) reported lack of adequate information about the content of the rehabilitation. Additionally, family member’s satisfaction with the care delivered decreased as they progressed from in-patient to community-based rehabilitation.

The importance of rehabilitation pathways with direct transfer from acute care in the Trauma centre to specialised rehabilitation is reported in two Norwegian studies; a direct transfer implies better functional outcomes for the patient and is more cost-effective in a long-term perspective [17, 18]. A recent systematic review exploring the experience of patients with acquired brain injuries (ABI) and their family members during the hospital stay, found that family experience included difficulty adjusting after the patient’s injury and a high need for information [19].

To address a gap in the relevant literature, a multidimensional scale including aspects of the family member’s satisfaction with and experience of health care services after TBI have recently been developed and validated [20]. The family experiences of care questionnaire in severe traumatic brain injury (FECQ-TBI) is measuring family members’ experiences from both the acute and rehabilitation phase after severe TBI [20].

A better understanding of the factors affecting a family member’s experience may assist health care providers in improving the quality of services in the future. To what extent family member demographic data and patient-related determinants affect the experience with the health care provided remains to be explored.

The aims of this longitudinal study were the following:

1. Assess family member experiences with care provided in the acute phase and during in-hospital rehabilitation using the FECQ-TBI.
2. Investigate the relationship between family member characteristics, patient demographics, patient dependency and rehabilitation pathway in relation to the family members experiences with care.

## Methods

### Design

This is a study about the family members experiences of clinical health care services provided within trauma hospitals and rehabilitation units. The study included family members of patients aged 16 years or older with severe TBI that occurred between January 2009 and December 2011. The included patients were admitted to one of the four trauma referral centres in each of the four health regions in Norway. Inclusion procedure for the patients in the national multicenter study on severe TBI was: Admitted to a trauma center in Norway within 72 h after injury, age 16 years or older, ICD-10 diagnoses codes corresponding to intracranial injuries (S06.0-S06.9), non-sedated Glasgow Coma Scale score ≤ 8 during the first 24 h post-injury. Exclusion criteria were: other chronic neurological diseases, severe psychiatric disease and/or severe alcohol/substance abuse or if they did not consent [21].

The inclusion criteria for the family members were as follows:

- Family member of a patient included in the national multicentre study on severe TBI.
- Age ≥ 18 years.
- The family member was listed as the patient’s closest relative either by the patient and/or in the patient’s medical records.

### Data collection

A close family member or acquaintance was identified by the regional project coordinator at each trauma centre. Written informed consent was required from both the person with the severe TBI and all participating family members. If the patient was not able to give consent due to cognitive impairments, the family member answered on his or her behalf. The project coordinator in each regional trauma centre collected this consent.

**Fig. 1:**
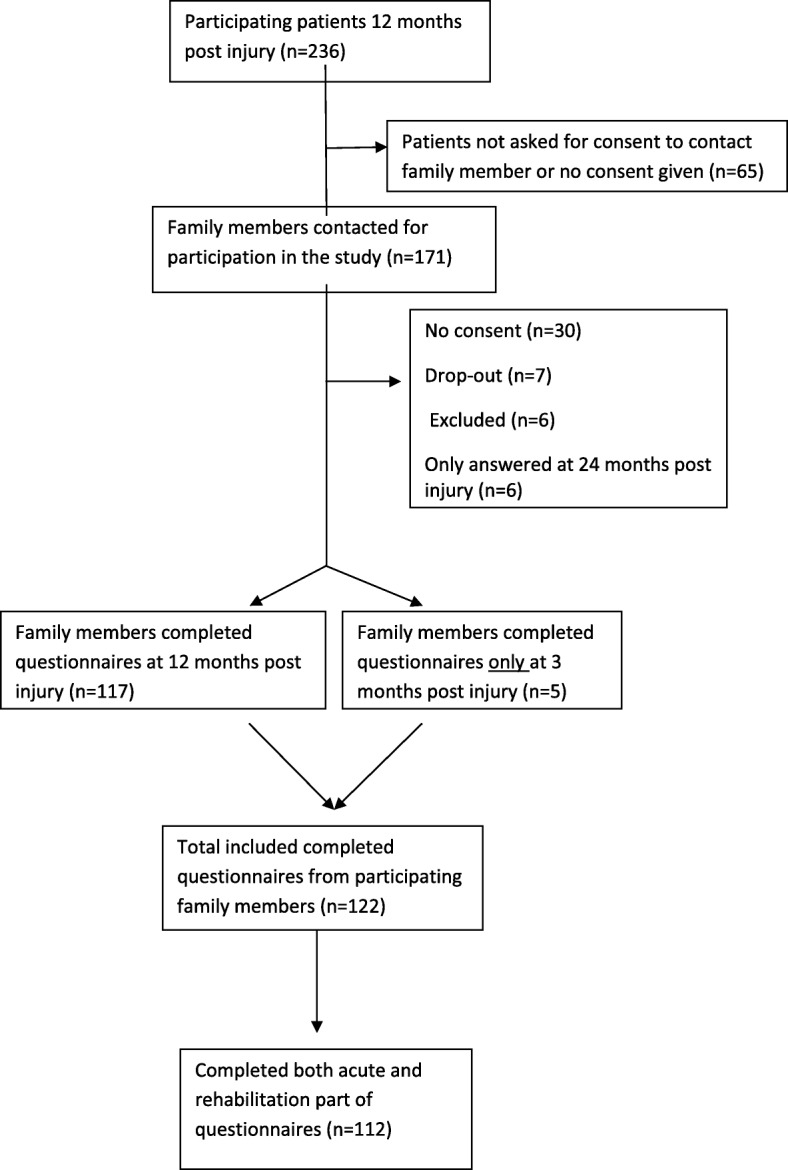
Flowchart of participating family members

Family members were contacted by telephone and/or letter and were given information about the study by a coordinator working at the University Hospital of North Norway, who was responsible for the database. Written information about the study, consent forms and questionnaires were sent by mail. All participants had the opportunity to contact the project coordinators if they had any questions. The study was approved by the regional Committee for Medical Research Ethics in South-East Norway. Data on patients were obtained from the national multicentre study on severe TBI [21]. Data were collected from family members 3 and 12 months after injury for patients injured in 2010 and only 12 months post injury for patients injured in 2009 and 2011. In the present study, data collected 12 months post injury were preferred, although we included those family members who had only answered at 3 months (*n* = 5) in the analyses (Fig. 1).

### Family member measures

Demographic variables included gender, marital status, relationship to the patient and level of education. The level of education was dichotomized into low (12 years or fewer) and high (13 years or more, i.e., college/university education).

To assess the family members’ experience with the health care services, the newly developed family experiences of care questionnaire after traumatic brain injury (FECQ-TBI) was applied [20]. The FECQ-TBI contains six subscales and 39 items: acute organization and information (10 items), rehabilitation organization (13 items), rehabilitation information (6 items), discharge (4 items), hospital facilities patient (4 items) and hospital facilities family member (2 items). The 10 items related to the acute phase can be divided into two subscales that corresponds to the rehabilitation items: acute organization (5 items; Chronbach’s alpha 0.89) and acute information (5 items; Chronbach’s alpha 0.94) [20]. In addition, 7 single-item questions were included: overall satisfaction (2 items), incorrect treatment (3 items), economic needs (1 item) and if children involved were taken care of (1 item).

The FEC-TBI has shown good construct validity and internal consistency with Cronbach’s alpha coefficients of 0.80–0-94 [20]. Each item was scored from 1 to 5, representing the worst and best family member experience, respectively. Each subscale within FEC-TBI contains different numbers of questions, leading to different range of scores. To compare the subscales, an index score was calculated using the sum score of each subscale divided by the total number of items.

### Patients’ measurements

Patient demographic variables recorded during the acute phase included age, gender, level of education and marital status. The level of education was dichotomized into low (12 years or fewer) and high (13 years or more, i.e., college/university education). The Glasgow Coma Scale score (GCS) [22] was used to assess the patient’s level of consciousness in the acute phase of the TBI, and the lowest GCS score within the first 24 h was registered. Acute injury severity was assessed and scored by the most commonly used and previously validated scale: the Abbreviated Injury Score (AIS) head [23].

Functional independence was determined by the regional study coordinators as the actual use of formal personal assistance at 12 months post-injury and was broadly defined as comprising self-care and areas requiring more seldom formal assistance, such as housekeeping or leisure activities. The use of personal assistance was classified into the following 5 categories: several times a day, once a day, once every 7 days, once every 14 days, never. This was further categorized into three: 1) daily, 2) between once every 7 days up to once every 14 days, and 3) requiring no help (never). In addition, the individual patient classification of functional independence was controlled against scores of the Glasgow Outcome Scale Extended (GOSE) at 12 months post-injury to increase validity [24].

In the present study, the patients’ rehabilitation pathway was characterized by the following four routes after discharge from the acute department at the trauma centre: 1) direct transfer to TBI specialized rehabilitation, 2) delayed transfer to TBI specialized rehabilitation, usually via a local hospital, 3) transfer to non-specialized rehabilitation, and 4) no rehabilitation [18]. The TBI specialized rehabilitation units have a defined responsibility for patients with severe TBI beginning in the early stages after trauma, i.e., when the patients are medically stable [18]. These units were integrated in hospital departments or a specialized rehabilitation hospital, and all units employed multidisciplinary teams consisting of a medical doctor (MD) specializing in physical medicine and rehabilitation, a nurse, an occupational therapist (OT), a physical therapist (PT), a psychologist/neuropsychologist, a speech therapist, and a social worker. All of the patients received therapy with higher intensity than in general rehabilitation units. The non-specialized rehabilitation units include geriatric or rehabilitation units in local hospitals. However, the staffs of these units (mainly nurses, PTs, OTs) have limited specific training in rehabilitation after severe TBI in the sub-acute stage.

### Statistics

Statistical analyses were performed using the Statistical Package for Social Sciences (SPSS) for Windows version 24.0. The descriptive data are presented as means (M) and standard deviations (SD) or as proportions of subjects within predefined categories. Cross-tabulations with χ^2^-tests were performed for nominal data. Independent sample t-tests or analysis of variance (ANOVA) were used to compare the means between 2 or more groups.

ANOVA was also used to investigate scores in the different subscales of FEC-TBI in relation to family member and patient variables. The least significant difference (LSD) post hoc test was applied. Non-parametric statistical analysis was applied when data were not normally distributed (Kruskal-Wallis test or Mann-Whitney U-test). Paired sample t-tests were used to investigate differences between experience in the acute and rehabilitation phases.

The following subscales of the FECQ-TBI were skewed in a positive direction: 1) acute organization and information and 2) rehabilitation organization. Statistical analysis of the FECQ-TBI was therefore performed with nonparametric methods. If there were 1 or 2 missing data point(s) in the FEC-TBI, data were replaced with the family members mean value on each subscale. Participants who had more than 2 missing data points in each subscale (or more than 1 on areas including ≤8 questions) were excluded. *P*-values less than 0.05 were considered statistically significant.

## Results

### Participants

As illustrated in Fig. 1, a total of 171 family members were identified and contacted for participation in the present study (Fig. 1). Hundred twenty-two family members completed the questionnaires, corresponding to a response rate of 71%. Out of these, 117 completed the questionnaire at 12 months post-injury and 5 at 3 months only. Nine patients did not receive post-acute rehabilitation. Three family members had missing data in the subscale acute organization or in acute information (*n* = 119). One family member had missing data in the rehabilitation subscales (*n* = 112).

Seventy-nine per cent of the participating family members were female, and 84% of the patients were male. Forty-one per cent were spouses/cohabitants of the patient, whereas 43% were parents (Table 1). The characteristics of non-participating family members are not available.

**Table 1:** Family member characteristics (total *N* = 122). Presented as number of cases and percentages (%)

Family member characteristics	N (%)
Female	96 (78.7)
Married/cohabitant^a^	94 (81.0)
*Relationship to patient*	
Spouse/cohabitant	50 (41.0)
Parent	52 (42.6)
Children	4 (3.3)
Siblings	11 (9.0)
Other	5 (4.1)
*Level of education*	*n = 121*
Low (≤ 12 years)	77 (63.6)
High (≥13 years)	44 (36.4)

^a^Cohabitant status means if the caregivers are married or cohabitant in general and includes caregivers married to the patient and those married to others

There were no statistically significant differences between the patient characteristics of the participating (*n* = 122) and non-participating groups (*n* = 114), except that the proportion of male patients was greater in the participating group (*p* < 0.05). Out of the 122 patients, the distribution between the four participating trauma centres in Norway were as follows: North (*n* = 20), Middle (*n* = 14), South-East (*n* = 79) and West (*n* = 9). Patient demographics and acute injury severity variables are presented in Table 2.

**Table 2:** Patient demographics and acute injury severity variables

Patient characteristics	Participating patients *N* = 122
Age (years), mean (SD)	39.7 (19.1)
*Gender n (%)*	
Male	103 (84.4)
Female	19 (15.6)
*Level of education (n = 112) n (%)*	
Low (≤12 years)	76 (63.4)
High (≥13 years)	36 (32.1)
Married/cohabiting n (%)	53 (43.8)
AIS Head, mean (SD)	4.2 (0.9)
GCS, mean (SD)^a^ Length of stay, acute (median, range) Length of stay, rehabilitation (median, range)	5.4 (1.9) 14,5 (2–56) 11,0 (0–86)

*GCS* Glasgow Coma Scale, *AIS* Abbreviated Injury Scale, *ISS* Injury Severity Scale, *LOS* Length Of Stay. ^a^Lowest value of GCS measured during the first 24 h post injury

### Overall satisfaction with care

The family members reported a high overall satisfaction with care. The two single questions about overall satisfaction revealed that 85% of the participants were overall satisfied (score 4–5) with the patient in-hospital care, treatment and rehabilitation (mean index score 4.36, SD 0.82). Seventy-nine per cent were overall satisfied (score 4–5) with how they were treated as a family member during the patient’s in-hospital stay (mean index score 4.23, SD 0.90).

### Experiences from the acute and rehabilitation phases

Table 3 shows scores of the ten identical items for the acute and rehabilitation phases (Organization and Information). Using paired samples t*-*tests, eight out of ten items had significantly higher scores (better experiences) in the acute phase compared with the rehabilitation phase. Examples of questions with marked differences are [20]: “To what extent do you think the personnel demonstrated thoughtfulness and care for *the patient?”;* “To what extent do you think the personnel seemed professionally competent?”; “To what extent do you think the personnel was interested in hearing your opinions as a relative?” and “To what extent do you think the personnel gave you information and explanations that you understood?”

**Table 3:** Family members` experience with care in the acute versus the rehabilitation phase. Only items with identical questions in the acute and rehabilitation phase are presented

Scale/item	Acute, mean (SD)	Rehabilitation, mean (SD)^a^	Mean Difference	*p*-value
Organization and Information (10 items), total mean (SD)	4.11 (0.77)	3.86 (0.85)	0.268	0.001
Fixed group of nurses	3.85 (0.99)	3.92 (0.98)	−0.054	0.633
Staff collaboration	4.21 (0.83)	4.01 (0.95)	0.207	0.041
Care/rehabilitation well planned	4.19 (0.95)	3.92 (1.00)	0.297	0.010
Thoughtfulness, care for patient	4.44 (0.71)	4.20 (0.88)	0.257	0.002
Seemed professionally competent	4.50 (0.72)	4.17 (0.94)	0.327	0.002
Took account of family situation	3.94 (1.02)	3.78 (1.03)	0.186	0.071
Thoughtfulness, care for family members	3.93 (1.03)	3.55 (1.19)	0.416	< 0.001
Interested in your opinions	3.72 (1.15)	3.47 (1.17)	0.283	0.008
Gave understandable information Information test, examination	4.17 (0.93) 4.09 (0.98)	3.89 (1.05) 3.61(1.10)	0.295 0.527	0.007 < 0.001

^a^Only including family members of patients who received rehabilitation (*n* = 113)

### Distribution of scores within the FEC-TBI

The subscale acute organization had the highest score (mean 4.24, SD 0.70), whereas the discharge subscale had the lowest score (mean 3.05, SD 1.10) (see Table 4). There were significantly higher scores reported on the organization subscale than the information subscale in both the acute and rehabilitation phases (*p* < 0.001). Within the discharge subscale one question about who to contact regarding problems after discharge (“Did you receive information about what you could do in the event of problems or unexpected events after your return home?”), and one question about information about the long-term consequences of TBI (“To what extent did you receive information about the short and long term consequences of head injuries?”), had particularly low mean scores (mean 2.81 and 2.99).

**Table 4:** Family members experience with health care in relation to patient dependency and rehabilitation pathway (mean, SD). A high score represents a better experience

Characteristics	*Acute Organization* *(n = 119)*	*Acute Information* *(n = 119)*	*Rehabilitation Organization* *(n = 112)*	*Rehabilitation Information* *(n = 112)*	*Discharge* *(n = 112)*
*All, mean (SD)*	4.24 (0.70)	3.93 (0.96)	3.91 (0.81)	3.46 (0.96)	3.05 (1.10)^**d**^
*Dependency (patient)*					
No help	4.29 (0.68)	3.97 (0.97)	**3.90 (0.77)** ^a*^	**3.46 (0.96)** ^**a**^	3.06 (1.17)
Every 3–14 d	4.23 (1.01)	4.00 (1.05)	**3.21 (1.01)** ^a*^	**2.58 (1.42)** ^**a**^	2.79 (1.39)
Daily	4.11 (0.68)	3.81 (0.92)	**4.09 (0.84)** ^a*^	**3.66 (0.88)** ^**a**^	3.09 (0.81)
*Rehabilitation pathway*					
Specialized, directly	4.21 (0.70)	3.93 (0.98)	4.00 (0.73)	**3.60 (0.91)** ^**a**^	**3.25 (1.10)** ^**b**^
Specialized, not directly	4.38 (0.68)	**4.13 (0.87)** ^a*^	3.93 (0.91)	3.47 (1.12)	**3.03 (1.14)** ^**c**^
Non-specialized rehab	4.02 (0.88)	**3.29 (1.04)** ^a*^	3.82 (0.98)	**2.91 (1.06)** ^**a**^	**2.27 (0.68)** ^**b, c**^
No rehabilitation	4.28 (0.57)	4.00 (0.73)	–	–	–

Bolded numbers indicates significant differences: ^a^*p* < 0.05, ^b^*p* < 0.01, ^c^*p* = 0.051, * = not significant after non-parametric test

^d^Significant differences between the subscale Discharge and each of the other subscales (all *p*’s < 0.001, paired samples t-test)

The subscales hospital facilities patient and hospital facilities family member had mean scores of 4.27 and 3.35, respectively, a significantly higher score related to facilities for the patient than the family member (*p* < 0.001). The questions (“What did you think about the following conditions in the rehabilitation ward?”) were related to: cleanliness, meals (for patient/relatives), peace and quiet (patient room), rest rooms and accommodation provisions for the relative (ref. AA). Family members’ experiences with restroom/accommodation and meals for family members had a mean score of 3.60 and 3.09 respectively. The single question regarding economic needs (“Were your economic needs taken care of?”) had the lowest score of all items within the FEC-TBI, with a mean score of 2.29.

### Experiences with health care in relation to caregiver- and patient-related variables

In the analyses, there were no significant differences in experience related to the family and patient demographic variables (gender, education, relation to patient and patient age). However, family members of patients with some dependency (needed help every 3–14 days) reported lower scores compared with family members of both very dependent and independent patients (see Table 4). This difference was significant within the subscale rehabilitation information (*p* < 0.05). Experiences related to the patient’s rehabilitation pathway exhibited a lower satisfaction in family members of patients who received non-specialized rehabilitation. These differences were significant within the subscales rehabilitation information (*p* < 0.05) and discharge (*p* < 0.01).

## Discussion

This study is, to the best of our knowledge, the first longitudinal study to explore family members of severe TBI patients experience with health care services throughout the patients’ in-hospital acute care and rehabilitation. The study found that family members were overall satisfied with the in-hospital care provided. A better experience with most aspects of the patient’s care in the acute phase was found compared with the rehabilitation phase whilst the discharge phase represented the worst experiences for the family members.

In this study, most family members were female and caring for a male patient. Fourty-one percent were married/cohabitant with the patient, whereas almost 43% was the patients’ parent. This is in accordance with other studies involving both family members and patients with severe TBI [25–27].

### Overall satisfaction

The overall satisfaction with treatment, care and rehabilitation of the patient was high among the majority of family members. The family members were also overall satisfied with how they were treated themselves during the hospital stay. These results are consistent with several other studies assessing overall satisfaction with health care [8, 28, 29]. A high satisfaction score is not necessarily a meaningful indicator of the experience with health care because satisfaction studies are likely to be less sensitive to specific problems in the quality of health care [8]. It should be recognized that this is not a clinical study and that family members only witness care for a portion of the treatment; thus, they are only partial observers, and their experiences may not necessarily reflect the true quality of the health care provided.

### Family members’ experience in the acute phase versus the rehabilitation phase

A significantly higher score related to care during the acute phase were reported, indicating that family members had most positive experiences with the care provided in the acute department. A possible explanation for this difference may be that as the patient moves from acute care to in-patient rehabilitation, the focus changes from survival to long-term outcome [12]. Along with the shift in focus, during the rehabilitation phase, the medical condition is stabilized, rehabilitation departments have fewer staff resources and there is less or no longer need of technological equipment for monitoring the patient. This may also explain why the family members experienced less thoughtfulness and care for the patient during rehabilitation compared to the stay in the acute department. Thus, these results do not necessarily mean that treatment is poorer in the rehabilitation phase. There may be several reasons why family members observe that staff collaboration and treatment plans appear to function better in the acute department compared with the rehabilitation department.

A qualitative study from Norway exploring stroke patients experience with the rehabilitation department describes that the patient experienced being a part of the rehabilitation community, but some of their relatives experience being somewhat excluded [30]. Further, Arntzen et al. describes some of the relatives experience as not being given training to cope with the patient or guidance on how to facilitate or stimulate the stroke survivor to active participate after discharge [30]. Although multidisciplinary teamwork and goal setting are valued methods in rehabilitative treatment, the use of practical training and guidelines is less developed in this setting than during acute care [31, 32].

The transition from acute to rehabilitation departments has previously shown to cause anxiety among family members [13]. Studies have also reported a lack of information in the rehabilitation phase regarding the content of the rehabilitation and the impact of a severe TBI [14, 15]. Dodek et al. pointed to the lack of information and organization as one of the main challenges for family members as patients move from the ICU to the neurosurgical departments or from the neurosurgical department to rehabilitation [33]. Family members may have unrealistic expectations related to both the extent of follow up and improvement of the patients’ functional and cognitive improvement. As an important part of rehabilitation is to activate and train the patient for the goal of independency, this information should be given to the family members several times during the in-hospital stay.

As indicated in another study, informing families and patients about their rights (regarding social services, insurance, vocational measures) during a dramatic life event is sometimes neglected [15]. In the present study, 40% of the family members reported that they either did not receive this information at all or received only a small amount of it. Additionally, 60% reported that their economic needs were not taken care of. One Norwegian study have explored the satisfaction and experience of patients or relatives including a question regarding the hospital facilities [3].They reported a good satisfaction with the hospital facilities. The family members in the present study found the facilities for the patients to be generally satisfying but the facilities supporting rest and accommodation for family members to be poor has not been previously reported. These results need to be taken with caution and explored in future studies.

Our findings may be important for health care providers in evaluating treatment programs in order to improve all aspects of care in the rehabilitation phase. In future studies, a comparison of the different acute departments as well as the rehabilitation departments is necessary. This will help us to see a trend in the family members experience in the different phases of the patients’ in-hospital pathway.

### Preparing for discharge to home

The present study found that family experiences during the discharge phase scored lowest of all the subscales within the FEC-TBI. Specifically, approximately half of the family members reported having poor experiences regarding; the provision of information, who to contact if any problems should arise after discharge, and information about the long-term consequences of TBI. The phase before discharge to home is crucial for both relatives and patients because they are preparing to live with the consequences of severe TBI and for the future. It is critical that family members are involved and supported during this phase, as has been highlighted in several other studies [12, 14, 15]. Several studies have described the experiences of family members of TBI/ABI patients in the transition phase from hospital to home, and a lack of information regarding both the patient’s outcome and the long-term consequences of TBI and the changed family roles are highlighted as primary concerns [14–16, 34, 35].

The discharge phase is described as a time of emotional adjustment, reorganization of the occupational roles, and preparation for the future [16, 34]. In addition, after discharge to home, many family members adapt a new caring role for which they may be unprepared for [36]. Increased levels of stress and anxiety are found among relatives during this phase, which highlights the importance of generating a thorough plan before discharge to home [15]. This result may be attributed to the fact that family members are faced with the long-term consequences of caregiving once returning to the community.

Turner and colleagues (2007) recommends prospective studies exploring the experiences of family members and individuals with TBI during the transition phase to obtain a more in-depth understanding of the process [34]. The present study may provide important information about current experiences with the discharge phase. Moreover, the magnitude of the problem should open up possibilities for improving the preparation for discharge from in-patient rehabilitation to home.

### Experiences with health care related to patient dependency and rehabilitation pathway

Family members of patients with moderate dependency reported poorer experience regarding the information provided in the rehabilitation department. This may be because moderate dependency results from cognitive problems which is not as visible in the rehabilitation phase. The number of family members in this group was low (*n* = 8), which may also impact the validity of this result.

Family members’ experience was significantly related to the patient’s rehabilitation pathway regarding the provision of information and preparations for discharge, with poorer experiences when treatment was given in a non-specialised rehabilitation unit than in a TBI-specialised rehabilitation unit. Studies describing the same patient population as the present study reported better functional outcome in patients being discharged directly from acute care to specialised rehabilitation, than in patients with non-directly transfer to specialised rehabilitation [17, 18, 37]. However, except for the discharge phase, in-hospital family experiences were not significantly better with a continuous specialised rehabilitation pathway. The findings are interesting, but they should be explored further in future longitudinal studies with larger numbers of participants.

### Consideration of methodology and design

The FEC-TBI is a validated questionnaire that was designed to explore family members experience with the care provided to TBI patients [20]. Because health care is a multidimensional service, it is important to measure experiences within different aspects of the health care in more detail to identify the determinants of quality of care [7]. In addition, there is the issue of social desirable bias when completing questionnaires addressing experiences and satisfaction. Instruments on family needs after TBI focus on health information together with support and involvement with care [38, 39]. These areas are all included in the subscale information in the FECQ-TBI [20]. Few others, if any, validated instruments probing this population’s experience with health care exist in the literature. Many of the instruments measuring experiences and/or satisfaction with health care are designed for patients rather than family members [3, 40].

### Study strengths and limitations

The present study is a prospective national multicentre study with the possibility to connect the parallel patient study. This design provides a unique opportunity to identify factors related to the experience of family members and increases the possibility of generalizing the findings. The longitudinal design and the use of a validated instrument are strengths of the study. Thompson’s review reported some concerns regarding studies of caregivers of patients with TBI: the variability in time since the injury, the use of non-validated instruments and the investigation of multiple injury severities to obtain a larger sample size [5].

Limitations of the study are as followed: the relatively low number of participants may result in low statistical power. To increase the number of participants, we also included those family members who only participated at 3 months post injury. This may have influenced the results. Indicators from acute and rehabilitation care that address functional status at discharge were not available. Additionally, a possible recall bias may be present due to the time span from patient discharge from the hospital to family member follow-up at 12 months post-injury. As this is an observational study, conclusions regarding causality cannot be established. Therefore, the results must be validated in future prospective studies of family members of patients with severe TBI. Additionally, the time of follow-up may need to be more connected to the patients’ discharge from the hospital to avoid the possibility of recall bias.

## Conclusion

This study provides detailed insight into the experiences of family members of patients with severe TBI in Norway. This work contributes to a better understanding of particular areas in the health care services provided after severe TBI that could be improved: 1) the poorest experiences were reported in relation to discharge, 2) experiences were better in the acute phase than in the rehabilitation phase, and 3) information regarding economical needs and consequences of the injury had low scores. The results must be taken with caution considering the different perspective and focus of the patient’s treatment and follow-up within the acute and rehabilitation phase respectively. However, the results are important for future clinical decision-making, the provision of health care, and the design of future health care policy and programmes to improve the services provided to family members and individuals with severe TBI.
